# Treatment for Moral Injury: Impact of Killing in War

**DOI:** 10.1007/s40501-022-00262-6

**Published:** 2022-05-07

**Authors:** Kristine Burkman, Rebecca Gloria, Haley Mehlman, Shira Maguen

**Affiliations:** 1 San Francisco VA Medical Center, 4150 Clement Street (116-E), San Francisco, CA 94121 USA; 2 University of California, San Francisco, CA USA

**Keywords:** Moral injury, Killing, Veterans, PTSD, Self-forgiveness, Moral repair

## Abstract

**Purpose of Review:**

Veterans who kill in war are at risk of developing negative mental health problems including moral injury, PTSD, spiritual distress, and impairments in functioning. Impact of Killing (IOK) is a novel, cognitive-behaviorally based treatment designed to address the symptoms associated with killing that focuses on self-forgiveness and moral repair through cultivation of self-compassion and perspective-taking exercises, such as letter writing, and active participation in values-driven behavior.

**Recent Findings:**

In a pilot trial assessing IOK, participants demonstrated a reduction in multiple mental health symptoms and improvement in quality-of-life measures, and they reported IOK was acceptable and feasible. Furthermore, trauma therapists have reported that moral injury is relevant to their clinical work, expressed a desire for additional training on the impact of killing, and identified barriers that make addressing killing in clinical settings challenging. Data are currently being collected in a national multi-site trial to examine the efficacy of IOK, compared to a control condition.

**Summary:**

IOK fills a critical treatment gap by directly addressing the guilt, shame, self-sabotaging behaviors, functional difficulties, impaired self-forgiveness, and moral/spiritual distress directly associated with killing in war. Typically provided following some initial trauma-processing treatment, IOK can be integrated in existing systems of trauma care, creating a pathway for a stepped model of treatment for moral injury.

## Introduction

Over the past decade, there has been renewed interest and scientific study of moral injury among combat veterans as returning servicemembers demonstrate a range of post-combat sequelae that are not fully captured by posttraumatic stress disorder (PTSD) [1]. Historically, the Veterans Health Administration (VHA) has focused on the assessment and treatment of PTSD, typically linking traumatic events involving life threat or witnessing horrific acts to specific symptoms that can be targeted in treatment. There are two evidence-based psychotherapies (EBPs) for PTSD currently endorsed by the VHA, Cognitive Processing Therapy (CPT), and Prolonged Exposure (PE). However, a meta-analysis examining the efficacy of such treatments found that 60–72% of combat veterans continue to meet diagnostic criteria for PTSD, and their functioning remains measurably compromised [2]. For some, it is not the fear associated with direct threat or witnessing horrific acts, it is *engaging in violence* (such as killing) that generates feelings of guilt, anger, and spiritual distress, which in turn increases risk of suicidal ideation and attempts, alcohol abuse, and other functional difficulties, even after controlling for general combat exposure [3–5, 6•]. Growing evidence in the moral injury literature suggests that we need to expand our framework beyond the traditional fear-based traumatic response that is the focus of existing EBPs for PTSD, to fully address the wounds of war [7].

Despite high rates of killing in war [8] and the associated outcomes [9–14], veterans are not routinely assessed for killing experiences. Such assessment could assist with prevention and treatment efforts. Veterans have reported they avoid talking about killing and moral injury, even in therapy, due to uncertainty about whether killing is an appropriate discussion topic and concern about judgment from providers who are largely civilians that are perceived as being unable to understand [5]. Therapists also avoid the topic due to concerns about stigma, fears of their own reactions, and feeling inadequately trained to address the topic of killing and associated moral/spiritual concerns [15•]. Given the negative mental health problems associated with killing in war and the barriers to broaching the topic clinically, this is a necessary area for further study.

## Treatment development

We began by examining the impact of killing through a mixed-method approach. Initial quantitative research examined mental health outcomes associated with killing in war, showing that those who killed in war were at increased risk for posttraumatic stress disorder (PTSD), alcohol abuse, suicide, and functional difficulties after returning home, even after adjusting for the impact of general combat [11–14]. We found that killing is a unique risk factor that is associated with poor mental health outcomes.

We then conducted focus groups with veterans of multiple war eras (Post-9/11, Persian Gulf, Vietnam, Korea, and WWII) inquiring about their experiences with killing, how killing impacted their lives post-deployment, and whether they believed killing experiences were addressed in existing PTSD treatments. Veteran feedback was qualitatively analyzed to determine common experiences, beliefs, and barriers to addressing moral injury in existing models of treatment [5]. Focus group findings also allowed us to develop the Killing Cognition Scale (KCS), a self-report measure assessing beliefs about killing in war that highlights prominent themes revealed in focus groups (e.g., guilt/shame, self-betrayal of morality, loss of spirituality, and self-condemnation). Additionally, participant feedback revealed ways existing EBPs failed to address the impact of killing and allowed us to develop an initial treatment protocol (6–8 sessions). This initial protocol, called Impact of Killing (IOK), was designed to follow EBPs for PTSD to ensure that individuals engaged in some trauma processing as an initial step that would help to prepare them for IOK.

We tested our initial treatment protocol in a randomized, controlled pilot study [16•] with combat veterans from multiple eras (Korea, Vietnam, Gulf, Post-9/11) who were diagnosed with PTSD, endorsed being distressed by killing or feeling responsible for the death of others, and received prior EBP for PTSD. Study providers were all licensed psychologists who had received specialized training in PTSD and met weekly with the treatment developer (Principal Investigator, Shira Maguen, PhD) for consultation and evaluation of fidelity through completion of session checklists highlighting key elements that needed to be addressed. An intent-to-treat analysis (*N* = 33) revealed that individuals in the IOK treatment group, compared to the waitlist condition, experienced a statistically significant improvement in PTSD symptoms (*p* = *0.033)*, general psychiatric symptoms (*p* = *0.0068)*, and specific items on quality-of-life functional measures. For example, veterans who received the treatment were more likely to endorse taking part in community events or celebrations (*p* = *0.0074)* and were more likely to confide personal thoughts and feelings to loved ones (*p* = *0.049)*. Anecdotally, multiple veterans described reconciling with adult children they had been estranged from for years, reaching out and apologizing to ex-partners for their behavior post-combat, volunteering, visiting meaningful sites (e.g., gravesite of fallen soldier, Vietnam), and even returning to places of worship after decades away as a result of participating in this pilot study. Assessment of the KCS showed that following completion of treatment, veterans who received IOK demonstrated statistically significant decreases in their level of distress around various killing-related beliefs such as “I deserve to suffer for killing” (*p* = *0.009)* and endorsed greater understanding of the factors that led to killing. Furthermore, those who received IOK reported that the treatment was acceptable and feasible.

To further examine the IOK treatment protocol, we collected qualitative feedback from all participants who completed the pilot study [17•] and surveyed ten trauma providers who reviewed the treatment to refine the protocol [15•]. Overwhelmingly, both veterans and providers indicated that IOK served a need not addressed by existing EBPs for PTSD, and they believed many veterans could benefit from the protocol. Veterans stated that the treatment should be longer and suggested that more time be spent on the area of self-forgiveness. We revised the protocol accordingly by expanding it to 10 sessions. Another consistent finding from our qualitative analysis of veterans’ and providers’ responses to the protocol was the importance of tailoring treatment to fit individual veterans’ specific needs. Morality, spirituality, and killing in war are profoundly personal in nature; although IOK offers a contained and structured framework, it allows providers to be flexible, creative, and collaborative in their approach to treatment.

We are currently conducting a multi-site, randomized, controlled trial to examine the efficacy of IOK when compared to Present-Centered Therapy (PCT) for PTSD. Given the use of PCT as a comparison treatment in the initial trials examining efficacy of EBPs for PTSD, we selected PCT to be the control condition. We modified PCT in the first two sessions to include general psychoeducation around moral injury and administration of the KCS, the measure we developed assessing beliefs around killing. Both IOK and PCT were originally intended to be administered face-to-face with participants, but due to restrictions on in-person treatment delivery during the COVID-19 pandemic, we shifted our delivery of treatment to include (almost exclusively) telehealth. While this shift was challenging in many ways, it has expanded recruitment and allowed us to enroll participants from parts of the country beyond the surrounding areas of our study sites.

## Treatment description

IOK is a cognitive-behaviorally based intervention that consists of ten sessions of weekly, individual psychotherapy lasting 60–90 min each facilitated by a therapist who has specialized training in traumatic stress. Ideally, veterans will have either participated in an EBP for PTSD or engaged in some form of trauma-focused psychotherapy prior to engaging in IOK. This is due partly to the brevity in which the cognitive-behavioral model is reviewed and the intensity of emotion associated with moral injury. Clinical judgment is key, and we have found that veterans tend to be more engaged and benefit more from treatment if they have prior therapy experience processing traumatic emotional material.

The first session orients veterans to what they should expect from IOK (see Table 1), which includes informing them of the range of emotions that may arise and importance of self-care and safety-planning to protect against self-harming or self-sabotaging behaviors. We also highlight the importance of collaboration with the therapist and explain that assignments in future sessions will be determined based on how the veteran responds to various measures, session content, and between-session assignments. Finally, we emphasize that IOK is designed to be a springboard for ongoing work toward moral repair outside of therapy. Therapists assess prior attempts the veteran has made on their own to heal from moral injury in this first session, both to identify potentially self-destructive tendencies *and* to reinforce and validate any gains made through previous therapy, spirituality, or personal pursuits. As best we can, we validate steps already taken and encourage veterans to view IOK as an opportunity to take stock and map out steps towards acceptance, self-forgiveness, and moral repair.

**Table 1:** Impact of killing (IOK) treatment session-by-session outline

Session	Description	Content
1	Pre-treatment evaluation	Assessment, past work, barriers to treatment, and coping skills
2	Common responses to killing	Physiology, instinctual decisions, initial reactions, emotions, behaviors, beliefs
3	Killing cognitions — part 1	Killing cognitions, meaning of killing
4	Killing cognitions — part 2	Maladaptive killing cognitions (cont.), behavioral activation, intro to acceptance
5	Acceptance and moral injury	Acceptance (cont.), role of self-betrayal in moral injury, related sequalae
6	Defining forgiveness	Defining forgiveness and self-forgiveness, barriers to self-forgiveness, and intro to forgiveness plan
7	Forgiveness — part 1	Areas of forgiveness, gaining perspective, forgiveness letters (cont.)
8	Forgiveness — part 2	Forgiveness letters (cont.), function of self-forgiveness, intro to amends plan
9	Taking the next step	Forgiveness letters (cont.), making amends, connection to others
10	Maintaining gains	Healing as a process, plan to continue work

Another important component of the first session is therapist administration of the KCS to the veteran while remaining nearby and available for reactions or questions. We believe this is a critical step based on feedback from both veterans [5, 17•] and providers [15•] regarding the challenge they experience in initiating discussions of killing. Therapists explain to patients that the items on the KCS reflect common thoughts and beliefs that combat veterans of all eras have shared; some may resonate, others may not. Based on their responses, treatment is tailored to focus on the areas of greatest conflict, distress, and/or functional impairment. Table 2 provides examples of the various categories and items captured in the KCS.

**Table 2:** KCS Categories and Example Cognitions

Category	Example Cognition
*Guilt*	“I had feelings that I should not have had at the time when I killed.”
*Shame*	“I worry about what my family and friends would think of me if they knew I killed someone.”
*Self-blame*	“I deserve to suffer for killing.”
*Responsibility*	“I was responsible for an unjustified killing.”
*Loss of meaning*	“Nothing seems important anymore after killing.”
*Contamination and self-loathing*	“I am forever tainted because of killing.”
*Remorse/regret*	“I wish I could have changed the outcome after seeing the humanity of the person I killed.”
*Moral violation*	“No good person would have done what I did.”
*Spiritual disillusionment*	“I wonder where God was when the killing happened.”
*Disgust/dismay*	“It bothers me that I felt a rush when I killed.”
*Other*	“I don’t trust my own anger after killing.”

In the second session, therapists provide information that other veterans have shared about their war experiences. We believe that this discussion of common reactions to killing may help to normalize their reactions to killing that they may perceive as being unique to them (and shameful). Common responses to killing include physiological responses, instinctual decisions, and the role of training in being able to kill and group dynamics of the unit that impact how individuals make sense of killing. Therapists also share common, potentially concerning emotions and beliefs that may emerge immediately after killing or much later. At the end of this session, a writing exercise is assigned which asks veterans to reflect on how killing has changed their view of themselves and others, as well as their sense of meaning and ability to function in life. Beliefs generated from this assignment, responses on the KCS, and reactions from the common responses to killing session are used to identify problem areas. Therapists focus on these areas to both challenge some beliefs with cognitive restructuring and to earmark others for acceptance, grief, and self-forgiveness work in the latter part of treatment.

The cognitive-behavioral framework is reviewed in the third session. Most veterans receiving IOK are familiar with it due to prior engagement in EBPs for PTSD. Here, therapists focus on the beliefs about killing that are most conducive to Socratic questions while acknowledging that sometimes as more details emerge, it becomes clear the thought may be better addressed through acceptance and self-forgiveness work. Veterans work with therapists in session and complete between-session assignments (e.g., thought records related to killing cognitions) to gain perspective, recognize the importance of context, and more fully integrate aspects of self that may be distorted or dismissed. Thought records for IOK include four columns (situation, cognition, emotion, behavior), with opportunities for documenting revised thoughts as appropriate.

In session 4, the importance of recognizing behaviors associated with reactions to killing cognitions is highlighted, as they often lead to seeking out or even creating confirmatory evidence to support self-condemning beliefs. We have found that veterans often interpret the self-destructive behavior that is a consequence of killing-related cognitions as further “evidence” that they are not a good person. To prepare for further sessions, it is imperative for veterans to understand how self-destructive behaviors contribute to long-held negative beliefs about themselves, others, and humanity, and to highlight how new behaviors can catalyze new beliefs that more fully reflect their true self.

While some beliefs related to killing can be challenged, others (i.e., appropriate guilt) may be accurate and need to be acknowledged and addressed in a different way. By session 5, therapy shifts away from cognitive restructuring and focuses instead on acceptance, self-forgiveness, and making amends. The fifth session acknowledges the range of consequences that veterans who have killed in war face (e.g., survivor guilt, self-condemnation, social isolation, suicidal ideation, self-harming and self-sabotaging behaviors, and spiritual disillusionments) and focuses more directly on the definition and concept of moral injury. There is also an acknowledgement that killing often happens in the context of other morally injurious events as well as profound loss (e.g., loss of innocence, death of fellow servicemember).

Therapists begin the conversation about forgiveness with an assignment asking the veteran to define self-forgiveness, share where they learned about it, and examine whether they apply the same standards of forgiveness towards themselves as they do towards others. Exploring veterans’ moral and spiritual development is essential in understanding the personal and cultural factors that influence moral injury *and* moral repair. It is common in the sixth session for veterans to reject the concept of self-forgiveness because they believe it equates to condoning actions they feel are wrong, or “letting themselves off the hook.” Others feel forgiveness (of self or others) is not possible without justice, often resulting in self-punishment. Others believe only the individual harmed or God can offer forgiveness and that self-forgiveness is akin to violating one’s faith. The goal of the sixth session is not to reach agreement on a universal definition of forgiveness, but rather to allow veterans to define it for themselves and take specific actions to better understand what is needed to move towards forgiveness of self and others. Through carefully understanding *how* veterans define forgiveness, *why* they apply forgiveness to others differently than they do themselves, and *what* specific barriers they identify as preventing self-forgiveness and forgiveness of others, veterans are able to develop a forgiveness plan with specific steps they can take to work through and with these challenges.

A powerful next step involves writing forgiveness letters in sessions seven through nine. The first letter is generally written to an individual killed, harmed, or not saved (e.g., with medics). Veterans are instructed to state the wrong they committed and their understanding of the impact of that wrong and to ask for forgiveness. Typically, these letters are to individuals who are dead or completely inaccessible (e.g., an unidentifiable civilian who was assaulted or killed during combat). The goal is to use the memory of these individuals and events to fully access emotions around the transgression, including the harmed individuals’ shared humanity (versus the dehumanization that often facilitates killing). The letters also help veterans to adopt and examine another’s perspective, which can alter ingrained ways of perceiving the identified morally injurious situation.

Additional letters are assigned to target different areas of forgiveness—for example, a letter from the perspective of a trusted other guiding the veteran on how to move forward in their life, and a letter to the pre-war version of oneself asking for forgiveness. We encourage therapists to work collaboratively with the veteran to ascertain whether writing additional letters (e.g., to family members of killed enemy soldiers or civilians, or loved ones that were harmed over the years by veterans’ self-destructive and isolative tendencies) would be helpful. Identifying the subject(s) of forgiveness letters is a collaborative process and involves the therapist’s clinical judgment. The point is to ensure that veterans are engaging in an authentic process of seeking forgiveness and the letters often reveal powerful themes of remorse, loss, grief, despair, and longing for change.

Letters are the cornerstone of the forgiveness plans; however, we also encourage other activities to explore the definition and function of self-forgiveness. These include generating a pros/cons list anchored on the function of self-forgiveness, various reading materials about forgiveness, consulting with the chaplaincy or other spiritual leaders, and/or practicing meditation or prayer. Veterans who completed the pilot trial shared that the forgiveness assignments were by far the most challenging part of treatment and yet also the most powerful [17•]. We have found that vigorous exploration of forgiveness and self-forgiveness often reveals deeply held, intact, moral values that therapists can help the veteran to actively honor. Letters are part of the therapeutic process rather than a product to share with others. Disclosure of killing experiences to others can be very complicated and is not an explicit goal of the treatment.

The final two sessions of the protocol focus on developing a plan for veterans to make amends. Making amends is a personal process that provides veterans with a chance to incorporate cultural ideas about self-forgiveness into a concrete action plan for atonement and continued healing post-treatment. Amends may involve engaging in commemorative or healing rituals, volunteering, or spending more time with family or loved ones. When the person or people wronged may not be available for direct amends or when a direct apology might create additional harm, the veteran can instead commit to living their life in a way that honors the spirit of what that amends might be (i.e., a “living amends”). Veterans in IOK are encouraged to live their lives in a way that honors their articulated morals and values, and the amends plan maps out specific steps that can be taken to continue to heal. Here, many veterans wrestle with whether or not to tell others about their killing experience. While disclosure is not a specific goal of IOK, it is important to help veterans examine their expectations and prepare for a range of reactions if they choose to share.

Because IOK is designed to be a springboard for ongoing healing, the last assignment is a reflection statement that asks veterans to assess what has shifted or changed since they started treatment. Therapists also specifically ask about areas that remain conflicted and how the veteran plans to continue working towards acceptance, self-compassion, and forgiveness. By acknowledging the ongoing/persistent nature of forgiveness and self-forgiveness, veterans are encouraged to accept that some aspects of their experiences may never be fully resolved. Cautioning that false or forced forgiveness is temporary and may only add to the wound of moral injury, therapists encourage greater and deeper connections with their spirituality/faith communities, social support systems, and loved ones to help support them as they continue to heal.

## Unique features of IOK

IOK is designed to complement and follow existing EBPs for PTSD, such as PE and CPT. Many veterans will benefit from these treatments, and yet for some, offering focused moral injury treatment might allow for further healing. Below, we outline some core features that are unique to IOK and offer something that is not always addressed in existing EBPs for PTSD.

### *Distortions vs. acceptance*

Exposure (both in vivo and imaginal) and Socratic questioning with trauma reactions are powerful interventions that can challenge erroneous relationships made in the aftermath of traumatic events. However, for some veterans, it is the clarity of our human capacity for destruction and cruelty that is most haunting. IOK acknowledges the need for acceptance and grief work around acts of commission or omission that cannot be changed and violated deeply held beliefs about right and wrong. IOK is explicit in the process of separating out beliefs that can be challenged to reveal a more balanced truth from those beliefs that need to be acknowledged as a painful reality and earmarked for a forgiveness and amends framework.

### Direct language

A major finding from the focus groups with combat veterans was how important they felt it was to use “killing” in our assessment and treatment of combat experiences [5]. Some veterans reported that they had been in trauma treatment for years, even decades, and had never been asked directly about killing in war which made them think (1) killing was not an appropriate topic for treatment, and/or (2) clinicians might judge them if they volunteered that information. In our interviews with trauma providers, there was unanimous agreement that the topic of killing was an appropriate and important issue in treatment. However, providers also shared that they were either explicitly taught *not* to ask about killing experiences given veterans’ report of insensitive and voyeuristic interactions with civilians, or they felt at a loss of how to broach the subject [15•].

We have found two approaches helpful in initiating a conversation about killing. First, embedding questions about killing or other morally injurious events in the context of general combat exposure is a good way to destigmatize the topic and recognize that killing and engaging in acts that some may find crossed a personally held moral line is part of what we ask of our servicemembers when we send them to war. Second, the development of the KCS allowed a shared language for providers and veterans to tackle this challenging topic. Veterans are informed that the items on the KCS were generated by combat veterans of multiple eras and that some items may resonate while others may not apply.

### Forgiveness

Another important difference in IOK is our use of forgiveness as a conceptual anchor. Self-forgiveness, which we focus on most in IOK, is an active process often rooted in veterans’ spiritual and/or moral upbringing, which is an area many providers express discomfort in addressing or report they have been trained to avoid as a topic of psychotherapy [18]. However, exploring veteran’s moral and spiritual development is vital to understanding personal and cultural factors that influenced the moral injury and often reluctance to pursue self-forgiveness. Providers must invite veterans into these often fraught conversations to learn what disturbs them so much about the concept of forgiveness and especially self-forgiveness.

Acknowledging barriers to self-forgiveness is key. For example, some veterans believe self-forgiveness equates to condoning actions they felt were wrong, or absolving themselves for something they should not, which might allow them to do it again. Other veterans think forgiveness (of self or others) is not possible without justice, therefore they serve as their own judge, jury, and at the extreme, executioner. IOK does not promise agreement on a universal definition of forgiveness; rather, it explores the concept to allow veterans to define it for themselves and take specific actions to better understand what is needed to move towards forgiveness of self and others.

### Catalyst vs. resolution

OK was designed to be a starting point for continued work outside the therapeutic relationship and assert that healing is an ongoing process requiring persistent, active participation on the part of the veteran to accept, forgive, and move forward in a way that honors their sense of morality. In recognition of the weighty existential nature of the questions veterans struggle with (e.g., human beings’ capacity for good and evil, karmic retribution, and redemption), it is critical that therapists using IOK offer space to lay out all the pieces contributing to the conflict without getting stuck in a rhetorical loop or conceding to a premature conclusion that ultimately rings hollow. It is in the naming of specific barriers to forgive oneself (e.g., not wanting to condone actions, fear it could happen again, desire for justice of those harmed/killed, and belief only God can grant forgiveness) that veterans reveal their core values often shaped by their moral and spiritual beliefs. By reflecting these concerns and the values they bely, and collaboratively developing a plan that identifies specific tasks, rituals, or participation in communities that veterans can *actively pursue*, therapists can help move the needle in the daunting task of moral repair.

In addition to IOK offering unique features that existing EBPs for PTSD lack, there are some important differences between IOK and emerging, evidence-based psychotherapies for moral injury. First, to be a good fit for IOK, veterans must endorse participating in killing or feeling responsible for the death of others *and* experience significant distress related to these events. Though we address many additional events that created moral injury in the course of IOK, we anchor much of the treatment on acts of perpetration (commission and omission). Another unique aspect of IOK is the prominence of the assessment measure that was developed closely with veterans to identify a range of beliefs that reflect moral injury related to killing. Responses on the KCS heavily influence providers’ conceptualization and course of treatment, which is highly tailored to the individual. While IOK has a strong focus on self-forgiveness, much like Griffin and colleagues’ self-forgiveness workbook [19], IOK is conducted in the context of individual psychotherapy and focused on acts of war vs. interpersonal betrayal. Similar to Trauma Guilt Reduction Therapy [20] and Adaptive Disclosure [21], we also focus on guilt and shame, and additionally there is a very strong focus on acceptance of moral transgressions and committed action focusing on moral repair. IOK involves multiple writing exercises that encourage veterans to explore several perspectives (i.e., to the individual killed/harmed, from a trusted other, to a younger version of themselves) to help cultivate self-compassion. Similar to Building Spiritual Strength [22], spirituality is also addressed in IOK, although the focus is guided by veterans’ responses to the KCS. Accordingly, in cases that a veteran denies profound spiritual distress, spirituality would not be a core focus of their treatment. As each emerging treatment for moral injury has its unique areas of focus, IOK is designed to serve veterans whose distress and functional impairment is associated with killing or feeling responsible for the death of others by focusing on self-forgiveness and cultivating self-compassion.

## Future directions

Combat veterans who have killed in war and experience moral injury, also commonly present with PTSD symptoms; however, there is evidence to suggest that depression, substance abuse, and other psychiatric conditions are also common following exposure to killing [3–5, 6•]. Additional research is needed to assess prevalence of moral injury among all veterans including those who do not meet criteria for PTSD and examine whether IOK could be as effective with those participants. We designed IOK to follow completion of EBPs for PTSD, but we have learned through conducting the multi-site trial that several veterans did not complete an EBP for PTSD, sometimes in part due to the intervention feeling invalidating of their experience (e.g., using Socratic questioning to challenge a belief reflecting appropriate guilt). We would like to examine whether IOK could be a stand-alone treatment vs. delivered following an EBP for PTSD.

For many veterans, the experience of killing made them feel excluded from humanity and they harbored beliefs for years or even decades about being less-than-human, or a “monster.” Although we specifically address the importance of integration into other communities outside of the VA (e.g., spiritual/faith communities, volunteer organizations, extended family), there is potential for more formal collaboration with community partners around the country. Having therapists with specialized training in moral injury and the impact of killing specifically can create a sufficiently safe space for initial work. However, it is crucial that the ten sessions described above are merely the start for veterans to reconnect and allow themselves to begin to see themselves in a more self-compassionate way. Self-compassion can be very elusive in isolation, so further development of ongoing programs to help veterans connect with others around activities that bring meaning and purpose may help ensure ongoing progress in the life-long pursuit of moral repair.

We are in the midst of a multi-site randomized controlled trial of IOK and are continuing to evaluate and refine the intervention. At this time, we are only training study therapists on the protocol. Similar to our initial pilot study, study therapists are independently licensed social workers and psychologists who all have specialized training in PTSD, specifically in EBPs for PTSD such as CPT and PE. Anecdotally, study therapists have shared that it has been helpful to have previous experience with EBPs for PTSD prior to learning IOK to facilitate nuanced conversations around appropriate guilt and remorse versus more traditional distorted beliefs around level of responsibility or lack of consideration of context. We look forward to being able to disseminate IOK more widely and offer additional training following completion of the current multi-site trial.

## Conclusions

Veterans who kill in war are at a uniquely high risk for suicide and a range of negative mental health problems [3–5, 6•] including moral injury. It is crucial that providers within the VA system and community-based settings assess for exposure to killing within a supportive environment and, in doing so, communicate an understanding that killing can be a part of the combat experience that creates significant distress and functional impairment for some. Emerging research on moral injury continues to highlight the need to expand our treatment options [1, 2]. Therapists who specialize in treating PTSD among combat veterans have reported that killing is a very important area of clinical focus and that they would benefit from additional information and training in this area. IOK fills a critical treatment gap by directly addressing the guilt, shame, self-sabotaging behaviors, functional difficulties, impaired self-forgiveness, and moral/spiritual distress directly associated with killing in war. It is imperative that treatments for moral injury go beyond the frame that current EBPs for PTSD offer, because although there is overlap between moral injury and PTSD, there are key differences between them that need to be directly addressed in treatment (e.g., appropriate guilt, self-forgiveness, spiritual disillusionment). Moral injury cuts across multiple domains, and treatments that foster moral repair should reflect the inherently multi-faceted nature of the wound.
